# Diet Quality Scores and Cardiometabolic Risk Factors in Mexican Children and Adolescents: A Longitudinal Analysis

**DOI:** 10.3390/nu14040896

**Published:** 2022-02-20

**Authors:** Abeer Ali Aljahdali, Karen E. Peterson, Alejandra Cantoral, Edward Ruiz-Narvaez, Martha M. Tellez-Rojo, Hyungjin Myra Kim, James R. Hébert, Michael D. Wirth, Libni A. Torres-Olascoaga, Nitin Shivappa, Ana Baylin

**Affiliations:** 1 Department of Clinical Nutrition, King Abdulaziz University, Jeddah 21589, Saudi Arabia; aaoaljahdali1@kau.edu.sa; 2 Department of Nutritional Sciences, University of Michigan, Ann Arbor, MI 48109, USA; eruiznar@umich.edu (E.R.-N.); abaylin@umich.edu (A.B.); 3 Department of Environmental Health Sciences, University of Michigan, Ann Arbor, MI 48109, USA; 4 Department of Health, Iberoamericana University, Mexico City 01219, Mexico; alejandra.cantoral@ibero.mx; 5 Center for Nutrition and Health Research, National Institute of Public Health, Cuernavaca 62100, Mexico; mmtellez@insp.mx (M.M.T.-R.); libniavib@gmail.com (L.A.T.-O.); 6 Center for Computing, Analytics and Research, University of Michigan, Ann Arbor, MI 48109, USA; myrakim@umich.edu; 7 Cancer Prevention and Control Program, Arnold School of Public Health, University of South Carolina, Columbia, SC 29208, USA; jhebert@mailbox.sc.edu (J.R.H.); wirthm@email.sc.edu (M.D.W.); shivappa@email.sc.edu (N.S.); 8 Department of Epidemiology and Biostatistics, Arnold School of Public Health, University of South Carolina, Columbia, SC 29208, USA; 9 Department of Nutrition, Connecting Health Innovations LLC, Columbia, SC 29208, USA; 10 College of Nursing, University of South Carolina, Columbia, SC 29208, USA; 11 Department of Epidemiology, University of Michigan, Ann Arbor, MI 48109, USA

**Keywords:** cardiometabolic risk factors, diet quality, inflammation, longitudinal analysis, population-based study, children and adolescent, Mexicans

## Abstract

There is limited evidence for the effects of diet on cardiometabolic profiles during the pubertal transition. We collected repeated measures of diet quality and cardiometabolic risk factors among Mexican youth. This analysis included 574 offspring of the Early Life Exposure in Mexico to Environmental Toxicants (ELEMENT) birth cohort followed up to three time points. Dietary Approaches to Stop Hypertension (DASH), alternate Mediterranean Diet (aMedDiet), and Children’s Dietary Inflammatory Index (C-DII^TM^) scores were computed from food frequency questionnaires. Higher DASH and aMedDiet scores reflect a higher diet quality, and lower C-DII scores reflect an anti-inflammatory diet. Cardiometabolic risk factors were lipid profile, glucose homeostasis, blood pressure, and waist circumference. Linear mixed models were used between quartiles of each diet score and outcomes. Compared to the first quartile, the fourth DASH quartile was inversely associated with log serum insulin (μIU/mL) [β = −0.19, *p* = 0.0034] and log-Homeostatic Model Assessment of Insulin Resistance [β = −0.25, *p* = 0.0008]. Additionally, log serum triglycerides (mg/dL) was linearly associated with aMedDiet score [β = −0.03, *p* = 0.0022]. Boys in the highest aMedDiet quartile had higher serum high-density lipoprotein cholesterol (mg/dL) [β = 4.13, *p* = 0.0034] compared to the reference quartile. Higher diet quality was associated with a better cardiometabolic profile among Mexican youth.

## 1. Introduction

The prevalence of childhood obesity is increasing worldwide. In the Latin America region, the prevalence for boys and girls aged 5–19 year increased from 1.6%, and 1.8% in 1975 to 10.4%, and 13.4% in 2016, respectively [1]. Childhood obesity is associated with increases in the risk and prevalence of cardiometabolic abnormalities [2,3,4,5]. The cluster of cardiometabolic abnormalities is a risk factor for the incidence of cardiovascular disease (CVD), cardiovascular-related mortality, all-cause mortality [6,7], and other chronic conditions in adulthood [8,9]. Targeting childhood obesity is crucial for effective primary interventions for “adulthood cardiometabolic sequela” [5], and understanding the determinants of cardiometabolic risk factors in youth can inform risk-reduction and prevention programs [4,10].

Diet is a well-established risk factor for cardiometabolic health [11]. Using dietary patterns to assess the association between diet and health outcomes has been suggested as superior to the traditional single-nutrient approach [12]. A dietary pattern summary score can be used to evaluate a subject’s overall diet and categorize their intake based on the degree of adherence to the eating recommendations used to construct the score [12,13]. This multi-dimensional approach allows for detecting the collective impact of multiple nutrients and delivering practical, holistic dietary messages [14,15], consistent with public health recommendations.

Evidence relating diet patterns to cardiometabolic health has identified three dietary scores relevant to pediatric populations. The Dietary Approaches to Stop Hypertension (DASH) and the alternate Mediterranean Diet (aMedDiet) are considered to have “the most evidence for CVD prevention” [16]. The DASH is an eating pattern for reducing blood pressure based on research findings sponsored by the US National Institutes of Health [17]. The aMedDiet is an eating pattern among people living in the countries bordering the Mediterranean Sea [18], which has shown favorable associations with obesity, cardiometabolic risk clustering [19], and cardiovascular health [20]. These two eating plans emphasize a higher consumption of fruits, vegetables, whole grains, and nuts [17,18]. The Dietary Inflammatory Index (DII^®^) is a tool to assess the inflammatory potential of the diet, and it has been associated with multiple inflammatory markers in adolescents [21,22] and adults [23,24,25,26,27]. The use of the DII in cardiometabolic health is well justified in light of the established link between inflammation and cardiometabolic abnormalities [28,29,30,31]. Contrasting the associations between each of these dietary scores and cardiometabolic risk factors is useful for accumulating evidence needed to formulate precise public health messages for preventing or managing cardiometabolic abnormalities in youth. Given that none of the three dietary scores were originally developed for Mexican youths, contrasting the associations is crucial to shed light on the role of eating habits, traditions, and cultural values in facilitating the adoption of these scores across different populations [32,33].

Current evidence about the associations between each of these diet quality scores and cardiometabolic risk in pediatric populations is inconsistent [10,34,35,36,37,38,39], underscoring the need for prospective cohort studies that investigate the relationship between diet quality and cardiometabolic risk factors [18,35,39,40,41]. Thus, the aim of this study was to investigate the relationship between diet quality scores, DASH, aMedDiet, and the Children’s Dietary Inflammatory Index (C-DII^TM^), and cardiometabolic risk factors using a repeated-measures longitudinal study design among Mexican youth enrolled in the Early Life Exposure in Mexico to Environmental Toxicants (ELEMENT) birth cohorts. We hypothesized that a lower diet quality and more pro-inflammatory diets will be associated with an impaired cardiometabolic profile, higher waist circumference, blood pressure and triglycerides (TG), impaired glucose homeostasis, and lower high-density lipoprotein cholesterol (HDL-C).

## 2. Materials and Methods

### 2.1. Study Population

The analytic sample consisted of children and adolescents from two of three cohorts comprising the ELEMENT project in Mexico City, Mexico [42,43,44]. A detailed description of the ELEMENT project has been published elsewhere [44]. In brief, during 1997–2004, 1012 mother-child dyads were recruited from prenatal clinics, which serve low- to middle-income populations [45]. At childbirth, mothers reported sociodemographic information. A sub-sample of mothers enrolled in Cohort 3 participated in a randomized controlled trial (RCT) of daily calcium supplementation (1200 mg) during their pregnancies up to one year postpartum [43,44]. Offspring were followed at multiple time points during childhood to collect relevant data about growth, diet, and health outcomes.

**Figure 1:**
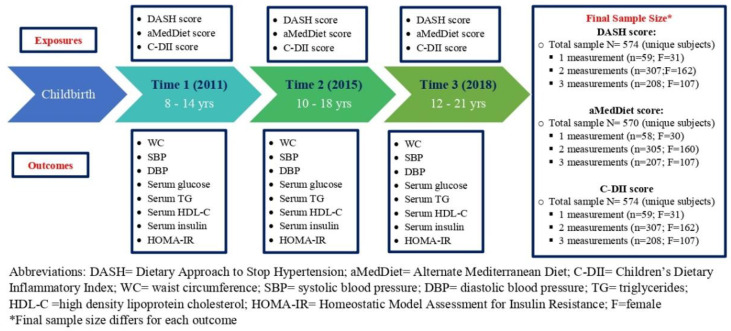
Flowchart summary of analytical samples of the Early Life Exposures in Mexico to ENvironmental Toxicants (ELEMENT) cohort.

The current analysis included 574 children and adolescents who attended at least one of three follow-up visits in late childhood and adolescence, had data for at least one of eight cardiometabolic risk factors (waist circumference, systolic and diastolic blood pressure, fasting glucose, fasting TG, fasting HDL-C, fasting insulin, and Homeostatic Model Assessment of Insulin Resistance (HOMA-IR)), and dietary information. At the 2011 follow-up visit, henceforth called Time 1, 250 children aged between 8–14 years were included [44]. Time 2, a follow-up study conducted in 2015, re-recruited 554 children aged 10–18 years [44]. In the 2018 visit, called Time 3, 518 adolescents aged 12–21 years completed the last follow-up visit. The sample sizes available and the number of repeated measures for each diet quality score are presented in Figure 1. The National Institute of Public Health of Mexico and the University of Michigan institutional review boards approved the research protocol (CI 599 and HUM00034344). The research team collected written informed consent and assent from mothers upon their enrollment and from adolescents, respectively.

### 2.2. Cardiometabolic Risk Factors

#### 2.2.1. Anthropometric Measures

Trained research staff collected duplicate measurements for body weight (kilograms [kg]) to the nearest 0.1 kg and height (centimeters [cm]) to the nearest 0.5 cm using in Time 1 a digital scale (BAME Model 420; Catálogo Médico/Tanita Co. Tokyo, Japan with height rod (model WB-3000m [38]), and only for weight in Time 2 and 3 the body composition device Inbody (model 230, Seoul, Korea). For waist circumference (cm), duplicate measurements were also performed to the nearest 0.1 cm using a non-stretchable measuring tape (SECA (model 201, Hamburg, Germany [38])). The average of the two measurements was used for the analysis [46].

#### 2.2.2. Cardiometabolic Biomarkers

For Time 1 study visit, duplicate readings for systolic and diastolic blood pressure were recorded with participants in a seated position using SpaceLabs 90217 Ambulatory Blood Pressure Measurement (Issaquah, WA, USA). Four cuff sizes: x-small (17–26 cm), small (24–32 cm), medium (32–42 cm), and large (38–50 cm), were available. For Time 2 and 3 study visits, duplicate readings for systolic and diastolic blood pressure were recorded with participants in a seated position using an automated blood pressure monitor (BPM-200 Medical Devices Blood Pressure Monitor, BpTRU; Coquitlam, BC, Canada). Following cuffs were available at these study visits: Child Cuff (13–18 cm), Adult-S (small: 18–26 cm), Adult-R (regular: 26–34 cm), Adult-L (large: 32–43 cm) and Adult-XL (Extra-large: 41–52 cm). Staff members assured the proper use of the cuff’s size based on the participant’s arm size. The average of the two measurements was used for the analysis. Fasting blood samples were used to analyze serum glucose via automated chemiluminescence immunoassay (Immulite^®^ 1000; Siemens Medical Solutions, Erlangen, Germany) [46], and TG and HDL-C using a biochemical analyzer (Cobas Mira Plus; Roche Diagnostics) [46]. Levels of insulin were quantified via enzyme-linked immunosorbent assay chemiluminescence method with Immulite ^®^ 1000; Siemens Medical Solutions, Erlangen, Germany [38]. A HOMA-IR was calculated as [fasting plasma glucose (mmol/L) × fasting serum insulin (mU/L))/22.5]; higher values represent lower insulin sensitivity/insulin resistance [47].

### 2.3. Diet Quality Scores

Dietary intake was assessed using a semi-quantitative food frequency questionnaire (FFQ) adapted from the nationally representative 2006 Mexican National Health and Nutrition Survey [48]. The FFQ contains 101 food items in 14 food groups: (1) dairy products; (2) fruits; (3) vegetables; (4) homemade fast food; (5) meat, sausages, and eggs; (6) fish and seafood; (7) legumes; (8) cereal and starchy vegetables; (9) corn products; (10) beverages; (11) snacks, sweets and desserts; (12) soups, creams, and pasta; (13) miscellaneous, and (14) tortillas. The FFQ queries usual intake over the previous week [38,48]. The frequency of consumption fell into 8 categories, ranging from never to 6 times a day [38]. Mothers of children younger than 11 years of age attended the study visit and assisted in the FFQ session to improve the accuracy and validity of children’s answers. FFQs were analyzed using a food composition software developed by the National Institute of Public Health, Mexico [49]. The average daily intake was calculated by multiplying the nutrient content for each food item by its frequency of reported consumption. Then, all intake values of all nutrients were summed to compute the daily consumption for each nutrient.

After grouping FFQ food items according to their nutritional properties, DASH and aMedDiet scores were calculated similarly to the methods proposed by Fung et al. (2008) [50] and Fung et al. (2005) [51], respectively (Supplementary Materials Tables S1 and S2). To account for the age and sex effects on dietary intake, we grouped our sample into 20 strata based on two-year increments by sex using a previously published approach [10,52] Starting with DASH score, the intake was ascendingly ranked into quintiles for each of eight components/food groups. Then, a score from 1–5 was given for each quantile. For each of the following components/food groups, fruits, vegetables, nuts and legumes, low-fat dairy products, and whole grains, we assigned 1 and 5 to quintile 1 and quintile 5, respectively. For sodium, red and processed meats, and sweetened beverages, we assigned 5 and 1 to quintile 1 and quintile 5, respectively. The component/food group scores were summed, and the possible range of scores was 8–40 [50]. aMedDiet score is the sum of eight indicators. For the fruits, vegetables, whole-grains, nuts, legumes, fish, and the ratio of monounsaturated to saturated fatty acids groups, if the intake was greater than the age and sex-specific median, a score of 1 was given. On the other hand, for the red/processed meats group, if the intake was less than or equal to the age and sex-specific median values, a score of 1 was given. Thus, the possible range of values was 0–8–down from 9 due to the exclusion of the alcohol group [51]. For DASH and aMedDiet scores, higher values indicate higher adherence to the diet pattern (i.e., individuals consumed more food/groups that characterized the dietary pattern).

Collected FFQ data at each time point was used to calculate the validated C-DII that included 25 components [53] (Supplementary Material Table S3). An inflammatory effect score was given to each C-DII sub-component according to their relationship with various inflammatory markers, which was based on published literature [23]. To calculate the z-score for each component of the C-DII score, each child’s dietary information was mapped to a population-based food consumption database composed of means and standard deviations from children in approximately 14 nations, which were referred to as global means and standard deviations [53]. The z-scores were calculated by subtracting the participants’ intake from the global means and dividing by the global standard deviations. The z-scores were standardized per 1000 calories to adjust for between-person variability in energy intake [54]. The scores were converted into centered percentiles by doubling the value and then subtracting 1 to minimize the right-skewing in the distributions. The resulting percentiles were multiplied by their corresponding inflammatory effect score to obtain a component-specific C-DII value. Lastly, each child’s C-DII score was the sum of its component-specific C-DII scores. The range of values for the C-DII in the current study was −4 to +4, where positive values indicate a more pro-inflammatory diet and negative values represent a more anti-inflammatory diet [38].

### 2.4. Covariates

Based on prior knowledge, potential confounders assessed for this research study were: (1) baseline characteristics assessed at childbirth or follow-up visit during the 5 years of child age, including sex, gestational age, mode of delivery, birth weight, duration of breastfeeding, and mother’s age, marital status, parity, years of education, and enrollment in the calcium supplementation RCT during pregnancy, and (2) follow-up characteristics for the children as measured at the follow-up study visits, including child’s age, body mass index (BMI), total daily caloric intake, physical activity measured as metabolic equivalents (METs), and pubertal status.

Mothers reported demographic information, including their age, marital status (married, or others—includes free union, single, separated, and divorced), parity status (1, 2, or ≥3), and years of education (<12 years., 12 years., or >12 years.), gestational age estimated by a registered nurse, mode of delivery (vaginal, or C-section childbirth), and enrollment in the calcium supplementation RCT (not enrolled, or enrolled). The newborns were followed until 5 years of age, and information reported by mothers about breastfeeding duration was quantified across the visits [55].

A physical activity questionnaire modified from the Youth Activity Questionnaire (YAQ) was validated relative to 24 hours physical activity recall among Mexican school-children aged 10 to 14 years [56]. The questionnaire was used to calculate total METs. For each self-reported physical activity; the corresponding MET [57] was multiplied by the activity intensity. The total METs per week was calculated by summing the METs for all activities. Tanner stages for sexual maturation were assessed by a trained pediatrician including female breast development, male genitalia and female and male pubic hair [58] with values ranging from 1 for pre-pubertal status to 5 for fully mature status [59,60]. In this study, pubertal onset was indicated by a value greater than 1 for one or more Tanner stages [61].

### 2.5. Statistical Analysis

The outcomes were (1) waist circumference (cm), (2) systolic and (3) diastolic blood pressure (mm Hg), (4) fasting glucose (mg/dL), (5) fasting TG (mg/dL), (6) fasting HDL-C (mg/dL), (7) fasting insulin (μIU/mL), and (8) HOMA-IR. Demographic characteristics of the study participants were presented as means (standard deviations (SDs)) for continuous variables and frequency (proportions) for categorical variables. To examine the correlation between the diet quality scores at each visit, we ran partial Spearman’s correlations adjusted for age, sex, and total caloric intake. Linear mixed-effects models with a compound symmetry error structure were used to examine the repeatedly assessed relationship between diet quality scores and each cardiometabolic risk factor. A generalized linear mixed model (specifically PROC GLIMMIX) with log links was used for the outcomes of TG, insulin, and HOMA-IR, as their residuals from the linear mixed-effects models indicated skewness. Residuals of the final models were assessed for the model assumptions. Diet quality scores were categorized into quartiles and median values were assigned to each quartile. Our models included quartile indicators of exposure, and the first quartile was considered a reference group. Additionally, we examined the linearity of trends across quartiles by modeling the quartiles as a continuous exposure variable. Findings are presented as β (standard error (SE)), and *p*-values (*p*).

The crude model included a variable for each diet score, and fully adjusted models included covariates that were considered potential confounders. Potential confounders were selected based on prior knowledge of the cardiometabolic health literature and their associations with the quartiles of each diet quality score. We had repeated measures for the following covariates: age, total daily caloric intake, physical activity (METs), and pubertal onset. All models were adjusted for total caloric intake and age, and sex only when models included boys and girls together. We also adjusted waist circumference models for BMI to account for body size [62]. In the tables, we present the results from the overall sample and sex-stratified models. To account for the multiple testing, a *p* < 0.00625 (0.05/8 [number of outcomes]) was considered a significant finding. The SAS statistical software package, version 9.4, was used for analyses (SAS Corp, Cary, NC, USA).

## 3. Results

Figure 1 summarizes the study design, sample sizes, and the number of repeated measures for each diet quality score. Table 1 shows the demographic characteristics of the youth and their maternal characteristics at childbirth stratified by study visit. The mean (SD) age of the sample was 10.32 (1.67), 14.50 (2.12), and 16.43 (2.14) years at Time 1, 2, and 3, respectively. Across the follow-up visits, the mean values of the cardiometabolic risk factors and diet quality scores varied. Time 1 had the highest values for the diet quality scores (i.e., higher DASH, and aMedDiet scores, and lower C-DII score (anti-inflammatory diet)); while Time 3 had the lowest diet quality scores (Table 1). The Spearman’s correlation coefficients [r_s_] between DASH and aMedDiet scores ranged from 0.39 to 0.45, for DASH and C-DII scores ranged from r_s_ = −0.53 to −0.57, and for C-DII and aMedDiet scores ranged from r_s_ = −0.43 to −0.47 across the three follow-up visits; all correlations were significant (*p* < 0.0001).

### 3.1. Association between DASH Diet Scores and Cardiometabolic Risk Factors

The distributions of potential confounding factors were examined across quartiles of the DASH diet score. DASH scores had medians of 19, 23, 26 and 29 in each quartile and were associated with several factors, including mother’s characteristics (such as enrollment in the calcium intervention study, parity, and years of education) and youth’s characteristics (such as pubertal onset and METs) (data not shown). In adjusted models, girls in the second DASH quartile had higher waist circumference (cm) [β = 1.11, *p* = 0.0041] compared to those in the lowest DASH quartile. An inverse association was detected with log serum insulin (μIU/mL) among participants in the highest DASH quartile compared to the lowest DASH quartile [β = −0.19, *p* = 0.0034], corresponding to a 19% reduction in serum insulin. Although the DASH score was linearly associated with log HOMA-IR [β = −0.02, *p* = 0.0050], corresponding to a 2.0% reduction for every unit increase in DASH score, the difference in log HOMA-IR between the DASH quartiles was significant only between the highest vs. lowest quartile with a 25.0% reduction [β = −0.25, *p* = 0.0008]. In the sex-stratified analysis, the inverse associations between log serum insulin and HOMA-IR were evident among boys only. No association was found with other cardiometabolic risk factors in the overall sample or the sex-stratified analysis (Table 2).

### 3.2. Association between aMedDiet Scores and Cardiometabolic Risk Factors

The aMedDiet scores had medians of 2, 3, 5 and 6 in each quartile. The aMedDiet quartiles were associated with several confounding factors, including mother’s characteristics (such as enrollment in the calcium intervention study, and mode of childbirth) and youth’s characteristics (such as pubertal onset and METs) (data not shown). In adjusted models, an inverse linear trend association was detected for log-serum TG (mg/dL) [β = −0.03, *p* = 0.0022]. This change represented a reduction by 3.0% in serum TG for every unit increase in aMedDiet score. Moreover, a positive association was detected with serum HDL-C (mg/dL) among boys in the highest quartile [β = 4.13, *p* = 0.0034] compared to the lowest quartile. No association was found with other cardiometabolic risk factors either in the overall sample or the sex-stratified analysis (Table 3).

### 3.3. Association between C-DII Scores and Cardiometabolic Risk Factors

The C-DII scores had medians of −1.809, −0.630, 0.367, and 1.627 in each quartile. The C-DII quartiles were associated with several confounding factors, including mother’s characteristics (such as enrollment in the calcium intervention study, parity, and years of education) and youth-related factors (such as pubertal onset and METs) (data not shown). In the fully adjusted models, no association was found between any cardiometabolic risk factors either in the overall sample or the sex-stratified analysis and C-DII scores (Table 4).

## 4. Discussion

In this longitudinal study, we examined the relationships between three diet quality scores and cardiometabolic risk factors among Mexican children and adolescents aged 8 to 21 years. Our study showed that insulin and HOMA-IR were inversely associated with the DASH scores, and TG was negatively associated aMedDiet scores. As far as we know, our study is one of a few prospective studies with repeated measures of multiple dietary quality scores and cardiometabolic risk factors among Mexican youth.

We found an inverse association between DASH score and HOMA-IR and serum insulin. Our results are consistent with findings from a meta-analysis of RCTs among adults [63], as well as a randomized cross-over clinical trial of 6 weeks of DASH intervention conducted among adolescent girls [64]. Moreover, the nutrients in the DASH diet have potential roles in insulin and glucose homeostasis [65,66,67]. The inverse associations with insulin sensitivity were of special interest for Hispanic youth because insulin resistance can occur in Mexican children without evidence of overweight or obesity [68]. Insulin sensitivity is a driver of adipose tissue partitioning [69], and abnormal fat deposition is a potent factor in the development and pathology of obesity [70]. Regarding the association between DASH score and blood pressure, our null results are consistent with other studies [37,71].

Our results showed that serum TG was linearly and inversely associated with the higher adherence to the aMedDiet pattern, which is consistent with the established role of diet in managing hypertriglyceridemia [72,73,74]. The inter-quartile increases were relatively small on serum TG (i.e., 3.0%) and may not be of clinical significance; however, a greater improvement in diet quality was associated with a higher effect size. This evidence collectively endorses controlling for serum TG as a potential primary intervention among youth to mitigate future cardiometabolic consequences, given the role of TG as an established risk factor for CVD among adults [75,76,77,78].

The positive association between HDL-C and aMedDiet scores was in agreement with intervention studies conducted among Mexican and Italian youth that showed positive associations between adhering to Mediterranean diet and serum HDL-C [34,79], as well as on HDL-C function (i.e., enhanced efflux capacity, reduced HDL-C oxidation) and quality (i.e., particles’ composition and size) [80].

We identified a few longitudinal studies conducted among Mexican youth with which to compare our results [38,81]. In a sub-sample of young adults in the ELEMENT cohort (N = 100, and mean age = 21.5 years), Betanzos-Robledo et al. examined the association between DII scores, as a cumulative exposure from the first year of life until 21 years of age; only blood pressure was positively associated with DII scores [38]. Moreover, Barragán-Vázquez et al. investigated the longitudinal association between C-DII scores and adiposity, assessed at 5, 7, and 11 years among Mexican children [81]. They found no association with waist circumference, which was consistent with our conclusions. However, a one-unit increase in the C-DII score was associated with a 0.41% change in waist circumference among girls [81]. Future longitudinal studies should examine the role of diet and cardiometabolic health in youth from different analytical perspectives [38,81].

One unexpected finding was a positive association between higher DASH score and waist circumference among girls. Waist circumference is an effective non-invasive tool for assessing truncal fat among children and adolescents [82]. However, repeated measures of waist circumference in childhood must be interpreted with caution as waist circumference captures information about subcutaneous fat, muscle, intramuscular fat, visceral fat, and bone [83]. The documented increase in waist circumference that parallels growth in children and adolescents [81,84,85] may not necessarily reflect a high-fat mass [84]. Additionally, waist circumference is affected by genetic and environmental factors [85], which may be a source of residual confounding.

Our sample had relatively lower diet quality and variability assessed by the three scores, which was consistent with other studies conducted on youth [86,87]. A plausible explanation could be that neither DASH or aMedDiet scores were developed to accommodate Mexican eating habits. Eating habits are influenced by culture [32], which is captured via methods of preparing foods, norms about food consumption, the availability of certain foods, and other factors [33]. Previously, it was shown that identifying empirically-driven dietary patterns did not necessarily capture the overall dietary pattern; rather, these patterns reflected the meal patterns within households among adolescents enrolled in the ELEMENT cohort [88]. In addition, no evidence was found to suggest a distinction between “westernized” or “traditional” patterns, as they were simultaneously incorporated into eating patterns among adolescents [88]; similar results were found in an adolescent Brazilian cohort [86]. This evidence showed the importance of considering the cultural context when assessing diet quality across different populations.

Differences in the associations between each diet score and cardiometabolic risk factors require explanation. We found moderate associations; others also have reported both moderate [89,90] and higher associations among diet quality scores [91,92]. The differences in the analytical methods deriving each score could be an additional reason [91,93]. Moreover, each score captures slightly distinct diet characteristics. We found that DASH score was associated with lower fat intake from all types. In contrast, aMedDiet and C-DII scores were positively associated with all types of fat, except for an inverse association for saturated fat and polyunsaturated fat (Data not shown). The DASH eating plan is characterized by reducing the intake of fat of all types [17] while aMedDiet and C-DII distinguish between fat types [18,94]

Our associations could have larger effect sizes if we had a longer duration of follow-up and greater variabilities in diet quality scores and cardiometabolic risk factors. Children and adolescents are generally metabolically healthy [95,96] and dietary exposures may require more time to manifest their impact on biomarkers of cardiometabolic health [97]. Further studies with longer follow-up duration are recommended to examine cardiometabolic abnormalities among youth as these associations may be pronounced in middle age.

The current study has several strengths. The ELEMENT birth cohort is a well-characterized cohort and permits adjustment for multiple confounders at baseline. We examined the overall associations in addition to sex-stratified associations due to the plausible differences among boys and girls in their eating patterns and their cardiometabolic profile during pubertal transition. Moreover, most other longitudinal studies limited their analysis to baseline dietary assessment in predicting the future occurrence of cardiometabolic risk factors [36,37]. Repeated assessment of dietary intake enhances our understanding of the change in dietary patterns during pubertal transition.

Despite its strengths, the current study has several limitations. The aMedDiet and the DASH scores use “population-specific” cut-offs for food consumption that allowed for these scores to be used in pediatric populations [10,52], despite their original application in adults [50,51]. Nevertheless, this may inflate type II error because of the reduction in diet variability in homogenous populations [98,99]. Another concern is that our sample may have had different scores if other cut-off values were used [33,93,100,101,102]. To address these limitations, we used C-DII scores as a third approach to assess diet quality. The C-DII scores use a population-based food consumption database from multiple countries as a reference [53,103]. The standardization of reference values in C-DII scores enhances cross-studies comparability and reduces the inherent bias that could occur when using the study population as a reference.

Moreover, dietary assessment in children and adolescents is subject to reporting errors due to limited skills in retrieving information or estimating portion sizes [104,105]. Diet quality patterns may not be a precise measure of overall healthy habits among adolescents [86,88] because they are not a comprehensive dietary assessment [106,107]. Also, the FFQs used in this study queried the intake in the previous week [105], which may not capture long-term dietary patterns, but could still be a reasonable estimation. Moreover, the FFQ did not measure eating behaviors, such as watching media while eating and unhealthy snacks between meals [108]. Another limitation is that the FFQ used has not been formally validated, but was used in the National Nutrition Survey of Mexico, which offered advantages of a culturally relevant food list [48]. It is worth noting that our conclusions may not be generalizable to youth with Mexican heritage who do not live in Mexico City due to differences in the regional and cultural context and available resources and assets. Lastly, the possibility of residual confounding could not be ruled out.

## 5. Conclusions

In conclusion, we found a protective association between higher diet quality and selected cardiometabolic risk factors, e.g., TG, HDL-C, insulin, and HOMA-IR among Mexican children and adolescents. Further studies are needed to validate the use of diet quality scores among youth and examine their reflection of the overall diet. Additional studies are warranted to enhance dietary assessments by including aspects of food habits and eating behaviors. Finally, healthy diet patterns may have a null or modest effect on cardiometabolic health outcomes compared to larger effect sizes for unhealthy eating patterns [109]. Thus, we endorse supplementing the diet quality assessment with indices of unhealthy eating behaviors, i.e., the consumption of processed foods, which is of great interest because Mexico had the highest annual retail sales *per capita* of ultra-processed food and drink products across Latin America [110,111], and the fourth highest worldwide [110].

## Figures and Tables

**Table 1:** Descriptive statistics of mother and child characteristics of the Early Life Exposures in Mexico to ENvironmental Toxicants (ELEMENT) analytical sample.

	Time 1 N = 250	Time 2 N = 554	Time 3 N = 518
Maternal Characteristics (at time of child’s birth)			
Years of education, %			
<12 years	123 (49.20) ^1^	284 (51.26) ^2^	265 (51.16) ^2^
12 years	91 (36.40) ^1^	187 (33.75) ^2^	171 (33.01) ^2^
>12 years	35 (14.00) ^1^	78 (14.08) ^2^	77 (14.86) ^2^
Age at childbirth, (years)	26.80 (5.63) ^1^	26.36 (5.40) ^3^	26.38 (5.44) ^3^
Parity, %			
1	93 (37.20) ^1^	209 (37.73) ^2^	194 (37.45) ^2^
2	89 (35.60) ^1^	194 (35.02) ^2^	183 (35.02) ^2^
≥3	67 (26.80) ^1^	146 (26.35) ^2^	136 (26.25) ^2^
Marital status, %			
Married	178 (71.20) ^1^	390 (70.40) ^4^	363 (70.08) ^4^
Others	71 (28.40) ^1^	157 (28.34) ^4^	148 (28.57) ^4^
Enrollment in calcium supplementation study, %			
Not enrolled	154 (61.60) ^1^	399 (72.02) ^2^	375 (72.39) ^2^
Enrolled	95 (38.00) ^1^	150 (27.08) ^2^	138 (26.64) ^2^
Child characteristics (at birth)			
Female, %	132 (52.80)	286 (51.62)	273 (52.70)
Gestation age, (weeks)	38.85 (1.49) ^5^	38.76 (1.61) ^6^	38.75 (1.60) ^6^
Mode of delivery, %			
Vaginal delivery	144 (57.60) ^7^	352 (63.54) ^8^	329 (63.51) ^8^
C-Section	103 (41.20) ^7^	194 (35.02) ^8^	181 (34.94) ^8^
Birth weight, (kg)	3.15 (0.45) ^9^	3.15 (0.49) ^4^	3.15 (0.48) ^4^
Breastfeeding duration, (months)	8.10 (5.88) ^1^	8.05 (6.07) ^2^	8.00 (5.98) ^2^
Child characteristics (at follow-up visits)			
Age, (years)	10.32 (1.67)	14.50 (2.12)	16.43 (2.14)
Body mass index, (kg/m^2^)	19.38 (3.60)	21.62 (4.15)	22.81 (4.46)
Body mass Z score for age	0.84 (1.24)	0.50 (1.25) ^8^	0.50 (1.25) ^10^
Pubertal onset, %	175 (70.00)	545 (98.38)	515 (99.42) ^11^
Metabolic equivalents, (METs/week)	31.39 (19.82)	57.23 (39.01)	44.95 (35.18) ^1^
Cardiometabolic risk factors			
Waist circumference, (cm)	70.75 (10.67)	79.56 (11.38)	85.53 (11.80) ^1^
Systolic blood pressure, (mmHg)	102.68 (10.20)	98.66 (9.92)	101.53 (9.83) ^1^
Diastolic blood pressure, (mmHg)	65.52 (7.32)	63.03 (6.86)	64.14 (7.20) ^1^
Glucose, (mg/dL)	87.02 (9.36)	77.81 (7.27) ^12^	90.22 (8.41) ^13^
TG, (mg/dL)	87.54(44.41)	103.97 (55.85) ^12^	105.52 (50.09) ^13^
HDL-C, (mg/dL)	58.68 (11.94)	43.06 (8.60) ^12^	44.70 (9.03) ^13^
Insulin, (μIU/mL)	6.26 (11.03) ^14^	19.06 (11.84) ^12^	19.21 (12.62) ^15^
HOMA-IR	1.59 (3.51) ^14^	3.69 (2.31) ^12^	4.32 (2.94) ^15^
Diet quality scores			
DASH diet scores	24.84 (4.06)	24.23 (3.99)	24.00 (4.00)
aMedDiet scores	4.26 (1.83)	3.81 (1.67)	3.77 (1.69)
C-DII scores	−0.16 (1.35)	−0.11 (1.43)	−0.10 (1.46)

Means (SD) or count (percentages) are presented for continuous or categorical variables, respectively. Number of missing values ^1^ n = 1; ^2^ n = 5; ^3^ n = 6; ^4^ n = 7; ^5^ n = 4; ^6^ n = 9; ^7^ n = 3; ^8^ n = 9; ^9^ n = 2; ^10^ n = 65; ^11^ n = 11; ^12^ n = 154; ^13^ n = 142; ^14^ n = 174; ^15^ n = 143. Abbreviations: TG = triglycerides; HDL-C = high density lipoprotein cholesterol; HOMA-IR = Homeostatic Model Assessment of Insulin Resistance; DASH = Dietary Approach to Stop Hypertension; aMedDiet = Alternate Mediterranean Diet; C-DII = Children’s Dietary Inflammatory Index.

**Table 2:** Linear mixed regression models for the relationship between quartile of dietary approaches to stop hypertension (DASH) score and cardiometabolic risk factors.

DASH Score ^1^		Waist Circumference (cm)			Systolic Blood Pressure (mmHg)			Diastolic Blood Pressure (mmHg)			Glucose (mg/dL)			Log TG (mg/dL)			HDL-C (mg/dL)			Log Insulin (μIU/mL)			Log HOMA-IR		
		All N = 574	Boys N = 274	Girls N = 300	All N = 574	Boys N = 274	Girls N = 300	All N = 574	Boys N = 274	Girls N = 300	All N = 435	Boys N = 213	Girls N = 222	All N = 435	Boys N = 213	Girls N = 222	All N = 435	Boys N = 213	Girls N = 222	All N = 410	Boys N = 202	Girls N = 208	All N = 410	Boys N = 202	Girls N = 208
		# obs. = 1297	# obs. = 621	# obs. = 676	# obs. = 1296	# obs. = 621	# obs.= 675	# obs. = 1296	# obs. = 621	# obs.= 675	# obs. = 1012	# obs.= 495	# obs.= 517	# obs. = 1012	# obs. = 495	# obs.= 517	# obs. = 1012	# obs. = 495	# obs.= 517	# obs. = 840	# obs. = 402	# obs.= 438	# obs. = 840	# obs. = 402	# obs.= 438
		**Crude model ^2^**																							
Quartile 1 Median = 19		**(Ref)**	**(Ref)**	**(Ref)**	**(Ref)**	**(Ref)**	**(Ref)**	**(Ref)**	**(Ref)**	**(Ref)**	**(Ref)**	**(Ref)**	**(Ref)**	**(Ref)**	**(Ref)**	**(Ref)**	**(Ref)**	**(Ref)**	**(Ref)**	**(Ref)**	**(Ref)**	**(Ref)**	**(Ref)**	**(Ref)**	**(Ref)**
Quartile 2 Median = 23	β SE	0.3290 0.7379	−1.7641 0.9053	2.6736 1.1428	−0.2851 0.7010	−1.1086 1.0205	0.5242 0.9425	0.1422 0.5197	−0.3087 0.7595	0.6257 0.7036	−0.01814 0.01028	−0.03199 0.01421	−0.00497 0.01462	−0.02739 0.03915	−0.03320 0.05055	−0.00404 0.05795	0.3166 0.9464	2.5288 1.3624	−1.7406 1.2842	−0.1135 0.06004	−0.1376 0.08008	−0.09521 0.08672	−0.1357 0.06520	−0.1861 0.08607	−0.09082 0.09513
	*p*-value	0.6557	0.0520	0.0197	0.6843	0.2778	0.5783	0.7844	0.6846	0.3741	0.0779	0.0249	0.7342	0.4843	0.5118	0.9445	0.7380	0.0640	0.1760	0.0591	0.0865	0.2730	0.0377	0.0312	0.3403
Quartile 3 Median = 26	β SE	0.09201 0.7617	−2.7801 0.9663	2.8635 1.1434	−0.6746 0.7160	−2.1720 1.0700	0.9136 0.9372	0.1368 0.5285	−0.8724 0.7908	1.2081 0.6986	−0.00357 0.01012	−0.01102 0.01431	0.003340 0.01406	−0.04297 0.04017	−0.08885 0.05455	−0.00079 0.05725	0.8016 0.9592	2.6308 1.4055	−1.0614 1.2775	−0.02292 0.05840	−0.1146 0.08184	0.04391 0.08160	−0.03110 0.06276	−0.1482 0.08715	0.05679 0.08886
	*p*-value	0.9039	0.0042 *	0.0125	0.3463	0.0428	0.3300	0.7958	0.2704	0.0842	0.7242	0.4417	0.8123	0.2851	0.1041	0.9891	0.4035	0.0618	0.4065	0.0591	0.1623	0.5908	0.6203	0.0899	0.5231
Quartile 4 Median = 29	β SE	−0.9829 0.8514	−2.5003 1.1382	0.5892 1.2309	−0.4748 0.7877	−0.3090 1.2243	−0.05971 0.9996	0.003925 0.5772	−0.6580 0.8948	0.8226 0.7432	−0.02344 0.01078	−0.02786 0.01572	−0.01465 0.01458	−0.07407 0.04518	−0.06178 0.06286	−0.08139 0.06342	1.0313 1.0494	3.4680 1.5760	−1.2203 1.3620	−0.1721 0.07142	−0.2227 0.1047	−0.1375 0.09639	−0.2218 0.07850	−0.2619 0.1118	−0.1895 0.1084
	*p*-value	0.2485	0.0285	0.6323	0.5468	0.8008	0.9524	0.9946	0.4624	0.2687	0.0300	0.0771	0.3153	0.1015	0.3262	0.1999	0.3260	0.0282	0.3707	0.0162	0.0340	0.1546	0.0048 *	0.0197	0.0810
Linear	β SE	−0.08280 0.08159	−0.2866 0.1078	0.07905 0.1193	−0.05908 0.07507	−0.1037 0.1157	0.01289 0.09584	0.002651 0.05492	−0.08367 0.08410	0.09677 0.07117	−0.00164 0.001019	−0.00197 0.001488	−0.00100 0.001378	−0.00708 0.004278	−0.00823 0.006007	−0.00661 0.005922	0.1084 0.1000	0.3329 0.1490	−0.09810 0.1309	−0.01183 0.006560	−0.01964 0.009345	−0.00729 0.008975	−0.01537 0.007096	−0.02392 0.01002	−0.01054 0.009819
	*p*-value	0.3104	0.0081	0.5077	0.4314	0.3705	0.8930	0.9615	0.3202	0.1744	0.1081	0.1873	0.4704	0.0985	0.1715	0.2652	0.2787	0.0259	0.4539	0.0718	0.0362	0.4173	0.0306	0.0174	0.2835
		**Adjusted model ^3,4,5^**																							
Quartile 1 Median = 19		**(Ref)**	**(Ref)**	**(Ref)**	**(Ref)**	**(Ref)**	**(Ref)**	**(Ref)**	**(Ref)**	**(Ref)**	**(Ref)**	**(Ref)**	**(Ref)**	**(Ref)**	**(Ref)**	**(Ref)**	**(Ref)**	**(Ref)**	**(Ref)**	**(Ref)**	**(Ref)**	**(Ref)**	**(Ref)**	**(Ref)**	**(Ref)**
Quartile 2 Median = 23	β SE	0.5597 0.2438	0.1253 0.2953	1.1055 0.3838	−0.3056 0.6906	−0.7790 1.0089	0.4733 0.9382	0.1631 0.5152	−0.07641 0.7473	0.6637 0.7018	−0.01824 0.01016	−0.02924 0.01417	−0.00462 0.01460	−0.02871 0.03882	−0.02728 0.04990	0.006651 0.05742	0.3541 0.7539	1.7154 1.0650	−1.3066 1.0386	−0.1192 0.05586	−0.1711 0.07680	−0.09504 0.08028	−0.1502 0.06118	−0.2224 0.08116	−0.1166 0.09012
	*p*-value	0.0219	0.6716	0.0041 *	0.6582	0.4403	0.6141	0.7517	0.9186	0.3447	0.0730	0.0396	0.7519	0.4597	0.5850	0.9078	0.6387	0.1080	0.2091	0.0332	0.0265	0.2372	0.0143	0.0065	0.1966
Quartile 3 Median = 26	β SE	−0.03468 0.2509	−0.3516 0.3155	0.3894 0.3828	−0.5741 0.7030	−1.6714 1.0605	0.9477 0.9303	0.1587 0.5221	−0.4328 0.7793	1.2149 0.6949	−0.00317 0.01002	−0.01045 0.01442	0.004289 0.01412	−0.04299 0.03956	−0.08448 0.05358	0.01517 0.05701	0.9758 0.7707	1.8873 1.1168	−0.2532 1.0372	−0.05021 0.05519	−0.1367 0.07799	0.000769 0.07804	−0.06758 0.05980	−0.1734 0.08134	−0.00826 0.08641
	*p*-value	0.8901	0.2657	0.3095	0.4143	0.1155	0.3087	0.7613	0.5789	0.0809	0.7520	0.4691	0.7615	0.2775	0.1156	0.7903	0.2058	0.0917	0.8073	0.3633	0.0805	0.9921	0.2588	0.0337	0.9239
Quartile 4 Median = 29	β SE	−0.01519 0.2811	−0.2061 0.3711	0.2409 0.4103	−0.1163 0.7730	0.08917 1.2070	0.1322 0.9899	0.1862 0.5695	−0.3518 0.8722	0.9279 0.7372	−0.02130 0.01076	−0.02664 0.01584	−0.01395 0.01466	−0.06989 0.04442	−0.05062 0.06156	−0.07330 0.06244	1.0360 0.8555	3.1918 1.2780	−0.9157 1.1101	−0.1943 0.06607	−0.3012 0.1027	−0.1310 0.08773	−0.2482 0.07341	−0.3569 0.1085	−0.1934 0.1006
	*p*-value	0.9569	0.5790	0.5574	0.8804	0.9411	0.8938	0.7437	0.6868	0.2086	0.0481	0.0933	0.3417	0.1160	0.4114	0.2410	0.2262	0.0128	0.4099	0.0034 *	0.0036 *	0.1363	0.0008 *	0.0011 *	0.0553
Linear	β SE	−0.01519 0.02697	−0.03387 0.03519	0.004382 0.03969	−0.02563 0.07361	−0.05473 0.1142	0.03158 0.09487	0.01777 0.05413	−0.04452 0.08211	0.1047 0.07058	−0.00144 0.001019	−0.00192 0.001505	−0.00091 0.001387	−0.00672 0.004210	−0.00729 0.005891	−0.00565 0.005834	0.1149 0.08184	0.2951 0.1214	−0.05378 0.1069	−0.01475 0.006133	−0.02550 0.009040	−0.00870 0.008334	−0.01893 0.006733	−0.03099 0.009520	−0.01338 0.009395
	*p*-value	0.5735	0.3361	0.9121	0.7277	0.6319	0.7393	0.7427	0.5878	0.1384	0.1571	0.2032	0.5108	0.1106	0.2166	0.3336	0.1608	0.0154	0.6153	0.0164	0.0050 *	0.2970	0.0050 *	0.0012 *	0.1550

^1^ Median values of DASH score at each quartile. ^2^ Model includes DASH score quartiles as fixed effects and compound symmetry matrix structure to model the covariance structure of the repeated measurements for each outcome. ^3^ Models additionally adjusted for the following fixed effects mother’s enrollment in the calcium intervention study, parity status, years of education at childbirth, child age, pubertal onset, metabolic equivalents, and calories. ^4^ Sex is an additional fixed effect in the adjusted models for the overall sample. ^5^ BMI is an additional fixed effect in the waist circumference models.* *p* < 0.00625.

**Table 3:** Linear mixed regression models for the relationship between quartile of alternate mediterranean diet (aMedDiet) score with cardiometabolic risk factors.

aMedDiet Score ^1^		Waist Circumference (cm)			Systolic Blood Pressure (mmHg)			Diastolic Blood Pressure (mmHg)			Log Glucose (mg/dL)			Log TG (mg/dL)			HDL-C (mg/dL)			Log Insulin (μIU/mL)			Log HOMA-IR		
		All N = 570	Boys N = 273	Girls N = 297	All N = 570	Boys N = 273	Girls N = 297	All N = 570	Boys N = 273	Girls N = 297	All N = 432	Boys N = 212	Girls N = 220	All N = 432	Boys N = 212	Girls N = 220	All N = 432	Boys N = 212	Girls N = 220	All N = 407	Boys N = 201	Girls N = 206	All N = 407	Boys N = 201	Girls N = 206
		# obs. = 1289	# obs. = 618	# obs. = 671	# obs. = 1289	# obs. = 618	# obs. = 670	# obs. = 1289	# obs. = 618	# obs.= 670	# obs. = 1006	# obs. = 492	# obs. = 514	# obs. = 1006	# obs. = 492	# obs. = 514	# obs. = 1006	# obs. = 492	# obs. = 514	# obs. = 835	# obs. = 400	# obs.= 435	# obs. = 835	# obs. = 400	# obs. = 435
		**Crude model ^2^**																							
Quartile 1 Median = 2		**(Ref)**	**(Ref)**	**(Ref)**	**(Ref)**	**(Ref)**	**(Ref)**	**(Ref)**	**(Ref)**	**(Ref)**	**(Ref)**	**(Ref)**	**(Ref)**	**(Ref)**	**(Ref)**	**(Ref)**	**(Ref)**	**(Ref)**	**(Ref)**	**(Ref)**	**(Ref)**	**(Ref)**	**(Ref)**	**(Ref)**	**(Ref)**
Quartile 2 Median = 3	β SE	−0.00397 0.7844	0.7594 0.9800	−0.9840 1.2059	0.4164 0.7559	1.3783 1.1285	−0.5512 0.9909	−0.08385 0.5620	−0.05524 0.8456	−0.07809 0.7386	−0.00357 0.01151	−0.00018 0.01632	−0.00761 0.01583	−0.00385 0.04129	0.005047 0.05501	−0.02006 0.05928	−0.2450 1.0179	0.7166 1.5139	−1.4923 1.3510	0.000894 0.06484	0.08904 0.09242	−0.06589 0.08965	−0.00519 0.07099	0.1151 0.09868	−0.1035 0.1005
	*p*-value	0.9960	0.4388	0.4149	0.5818	0.2225	0.5783	0.8814	0.9479	0.9158	0.7563	0.9914	0.6311	0.9258	0.9270	0.7351	0.8099	0.6362	0.2699	0.9890	0.3360	0.4628	0.9417	0.2442	0.3036
Quartile 3 Median = 5	β SE	−0.2745 0.7210	0.02010 0.9054	−0.6808 1.1045	0.2544 0.6813	2.1387 1.0115	−1.7062 0.8957	−0.2516 0.5025	0.4121 0.7494	−0.9031 0.6661	−0.01308 0.009827	−0.01314 0.01400	−0.01469 0.01345	−0.06952 0.03794	−0.03186 0.05027	−0.1064 0.05467	−0.2306 0.9057	1.2595 1.3255	−1.8469 1.2221	−0.03687 0.05918	0.06432 0.08509	−0.1130 0.08125	−0.05262 0.06449	0.06291 0.09156	−0.1444 0.08958
	*p*-value	0.7035	0.9823	0.5379	0.7089	0.0349	0.0572	0.6167	0.5826	0.1756	0.1834	0.3482	0.2756	0.0672	0.5266	0.0522	0.7991	0.3425	0.1314	0.5334	0.4502	0.1649	0.4148	0.4924	0.1077
Quartile 4 Median = 6	β SE	−2.2631 0.9080	−1.6717 1.1817	−3.0287 1.3487	0.1265 0.8487	0.4668 1.2956	−0.2324 1.0866	0.1132 0.6229	−0.5120 0.9521	0.7676 0.8072	−0.00235 0.01160	−0.01952 0.01708	0.01022 0.01546	−0.1193 0.04854	−0.08361 0.06650	−0.1565 0.06809	4.0263 1.1043	4.9457 1.6492	2.8388 1.4559	−0.1290 0.07998	0.02699 0.1122	−0.2499 0.1114	−0.1118 0.08538	−0.01289 0.1232	−0.1937 0.1163
	*p*-value	0.0128	0.1578	0.0251	0.8815	0.7187	0.8307	0.8558	0.5910	0.3420	0.8397	0.2536	0.5089	0.0141	0.2093	0.0220	0.0003 *	0.0028 *	0.0518	0.1073	0.8100	0.0254	0.1906	0.9167	0.0965
Linear	β SE	−0.3730 0.1952	−0.2823 0.2557	−0.4632 0.2881	0.02046 0.1813	0.3241 0.2771	−0.2624 0.2326	−0.01863 0.1329	0.008106 0.2023	−0.02874 0.1732	−0.00207 0.002493	−0.00511 0.003611	0.000150 0.003373	−0.02878 0.01019	−0.01741 0.01398	−0.03914 0.01431	0.6025 0.2403	0.8655 0.3553	0.3395 0.3192	−0.02406 0.01609	0.006684 0.02284	−0.04782 0.02216	−0.02430 0.01743	−0.00129 0.02443	−0.04287 0.02429
	*p*-value	0.0563	0.2700	0.1085	0.9101	0.2426	0.2597	0.8885	0.9681	0.8682	0.4075	0.1574	0.9645	0.0048 *	0.2137	0.0064	0.0123	0.0152	0.2881	0.1353	0.7700	0.0315	0.1635	0.9579	0.0783
		**Adjusted model ^3,4,5^**																							
Quartile 1 Median = 2		**(Ref)**	**(Ref)**	**(Ref)**	**(Ref)**	**(Ref)**	**(Ref)**	**(Ref)**	**(Ref)**	**(Ref)**	**(Ref)**	**(Ref)**	**(Ref)**	**(Ref)**	**(Ref)**	**(Ref)**	**(Ref)**	**(Ref)**	**(Ref)**	**(Ref)**	**(Ref)**	**(Ref)**	**(Ref)**	**(Ref)**	**(Ref)**
Quartile 2 Median = 3	β SE	−0.1404 0.2602	0.03642 0.3208	−0.3207 0.4021	0.5814 0.7470	1.4833 1.1171	−0.3454 0.9877	−0.05008 0.5595	0.02250 0.8350	−0.05735 0.7394	−0.00098 0.01132	0.001653 0.01611	−0.00258 0.01568	−0.01424 0.04090	−0.00912 0.05498	−0.03828 0.05885	0.1217 0.8142	1.7105 1.1721	−1.4624 1.0979	−0.01011 0.06014	0.1039 0.08814	−0.05645 0.08246	−0.01370 0.06598	0.1340 0.09244	−0.09206 0.09332
	*p*-value	0.5896	0.9097	0.4256	0.4366	0.1848	0.7267	0.9287	0.9785	0.9382	0.9310	0.9183	0.8691	0.7277	0.8684	0.5157	0.8812	0.1453	0.1836	0.8666	0.2392	0.4941	0.8356	0.1482	0.3245
Quartile 3 Median = 5	β SE	−0.3892 0.2458	−0.4985 0.3045	−0.3532 0.3777	0.4154 0.6912	2.3554 1.0291	−1.4118 0.9170	−0.2620 0.5134	0.4335 0.7584	−0.8423 0.6846	−0.00785 0.01002	−0.00725 0.01441	−0.00760 0.01375	−0.08973 0.03858	−0.05247 0.05128	−0.1248 0.05545	−0.00216 0.7566	2.2068 1.0793	−2.2225 1.0264	−0.03920 0.05608	0.04363 0.08402	−0.09581 0.07563	−0.05244 0.06160	0.04249 0.08899	−0.1275 0.08504
	*p*-value	0.1136	0.1023	0.3501	0.5479	0.0224	0.1242	0.6099	0.5678	0.2190	0.4331	0.6151	0.5805	0.0203	0.3069	0.0249	0.8812	0.0415	0.0309	0.4847	0.6039	0.2060	0.3948	0.6333	0.1345
Quartile 4 Median = 6	β SE	0.1856 0.3214	−0.3103 0.4056	0.4716 0.4887	0.4930 0.8973	1.2086 1.3530	0.06617 1.1824	0.2122 0.6643	−0.1003 0.9897	0.7708 0.8820	0.007172 0.01254	−0.01295 0.01842	0.02358 0.01711	−0.1316 0.05127	−0.09615 0.06949	−0.1723 0.07298	1.8205 0.9718	4.1344 1.4027	−0.3810 1.3088	−0.06270 0.07777	0.03936 0.1130	−0.1385 0.1071	−0.03106 0.08427	0.009474 0.1220	−0.06554 0.1154
	*p*-value	0.5638	0.4446	0.3350	0.5828	0.3721	0.9554	0.7495	0.9193	0.3825	0.5674	0.4825	0.1688	0.0104	0.1672	0.0186	0.0614	0.0034 *	0.7711	0.4204	0.7279	0.1966	0.7126	0.9381	0.5703
Linear	β SE	−0.03578 0.06906	−0.1396 0.08783	0.03408 0.1043	0.08428 0.1912	0.4670 0.2898	−0.2201 0.2515	−0.01518 0.1414	0.06694 0.2103	−0.04865 0.1880	−0.00025 0.002693	−0.00331 0.003920	0.002248 0.003706	−0.03302 0.01077	−0.02147 0.01470	−0.04273 0.01533	0.2389 0.2097	0.8033 0.3041	−0.2907 0.2818	−0.01457 0.01600	0.003412 0.02338	−0.03102 0.02186	−0.01332 0.01759	−0.00353 0.02468	−0.02637 0.02466
	*p*-value	0.6045	0.1127	0.7440	0.6594	0.1076	0.3818	0.9145	0.7504	0.7959	0.9251	0.3995	0.5444	0.0022 *	0.1449	0.0055	0.2548	0.0085	0.3027	0.3628	0.8840	0.1567	0.4491	0.8864	0.2856

^1^ Median values of aMedDiet score at each quartile. ^2^ Model includes aMedDiet score quartiles as fixed effects and compound symmetry matrix structure to model the covariance structure of the repeated measurements for each outcome. ^3^ Models additionally adjusted for the following fixed effects mother’s enrollment in the calcium intervention study, parity status, mode of childbirth, child age, pubertal onset, metabolic equivalents, and calories. ^4^ Sex is an additional fixed effect in the adjusted models for the overall sample. ^5^ BMI is an additional fixed effect in the waist circumference models. * *p* < 0.00625.

**Table 4:** Linear mixed regression models for the relationship between quartile of children’s dietary inflammatory index (C-DII) and cardiometabolic risk factors.

C-DII Score ^1^		Waist Circumference (cm)			Systolic Blood Pressure (mmHg)			Diastolic Blood Pressure (mmHg)			Log Glucose (mg/dL)			Log TG (mg/dL)			HDL-C (mg/dL)			Log Insulin (μIU/mL)			Log HOMA-IR		
		All N = 574	Boys N = 274	Girls N = 300	All N = 574	Boys N = 274	Girls N = 300	All N = 574	Boys N = 274	Girls N = 300	All N = 435	Boys N = 213	Girls N = 222	All N = 435	Boys N = 213	Girls N = 222	All N = 435	Boys N = 213	Girls N = 222	All N = 410	Boys N = 202	Girls N = 208	All N = 410	Boys N = 202	Girls N = 208
		# obs. = 1297	# obs. = 621	# obs. = 676	# obs. = 1296	# obs. = 621	# obs. = 675	# obs. = 1296	# obs. = 621	# obs. = 675	# obs. = 1012	# obs. = 495	# obs. = 517	# obs. = 1012	# obs. = 495	# obs. = 517	# obs. = 1012	# obs. = 495	# obs. = 517	# obs. = 840	# obs. = 402	# obs. = 438	# obs. = 840	# obs. = 402	# obs. = 438
		**Crude model ^2^**																							
Quartile 1 Median = −1.809		**(Ref)**	**(Ref)**	**(Ref)**	**(Ref)**	**(Ref)**	**(Ref)**	**(Ref)**	**(Ref)**	**(Ref)**	**(Ref)**	**(Ref)**	**(Ref)**	**(Ref)**	**(Ref)**	**(Ref)**	**(Ref)**	**(Ref)**	**(Ref)**	**(Ref)**	**(Ref)**	**(Ref)**	**(Ref)**	**(Ref)**	**(Ref)**
Quartile 2 Median = −0.630	β SE	−1.7767 0.7480	−1.5876 0.9488	−1.6627 1.1287	−0.8237 0.7132	−0.8569 1.0841	−1.0464 0.9262	−0.8375 0.5289	−1.2088 0.8073	−0.5412 0.6915	0.01185 0.01037	0.01802 0.01502	0.006933 0.01401	0.02667 0.04125	0.05751 0.05467	0.01242 0.05944	1.2633 0.9442	1.4994 1.4080	1.1387 1.2547	−0.01112 0.06234	0.02392 0.08838	−0.03165 0.08618	−0.00900 0.06819	0.04720 0.09667	−0.04518 0.09444
	*p*-value	0.0177	0.0950	0.1413	0.2484	0.4296	0.2590	0.1136	0.1349	0.4341	0.2536	0.2306	0.6209	0.5181	0.2935	0.8346	0.1812	0.2875	0.3646	0.8585	0.7868	0.7137	0.8951	0.6256	0.6326
Quartile 3 Median = 0.367	β SE	−0.7154 0.7746	0.9719 0.9874	−2.3282 1.1678	−0.01267 0.7302	0.8179 1.1069	−1.0997 0.9509	−0.3229 0.5389	0.006068 0.8185	−0.7512 0.7087	0.01915 0.01040	0.03382 0.01487	0.003251 0.01429	0.06592 0.04183	0.05300 0.05595	0.09213 0.05973	0.3462 0.9700	0.1860 1.4195	0.5931 1.3192	0.02611 0.06237	0.1313 0.08391	−0.06484 0.09243	0.03706 0.06781	0.1720 0.09130	−0.07919 0.1010
	*p*-value	0.3559	0.3255	0.0467	0.9862	0.4603	0.2479	0.5492	0.9941	0.2895	0.0660	0.0234	0.8201	0.1154	0.3441	0.1236	0.7212	0.8958	0.6532	0.6756	0.1184	0.4834	0.5849	0.0604 *	0.4336
Quartile 4 Median = 1.627	β SE	−0.4730 0.8139	0.5592 1.0580	−1.3017 1.2065	0.3410 0.7557	1.0353 1.1543	−0.5672 0.9737	−0.1045 0.5543	0.06080 0.8452	−0.3924 0.7246	0.009471 0.01064	0.02123 0.01554	−0.00136 0.01427	0.09871 0.04350	0.1510 0.05866	0.05567 0.06214	0.4201 1.0196	0.8044 1.5123	0.4001 1.3566	−0.04730 0.06760	−0.07156 0.09781	−0.01034 0.09126	−0.05217 0.07365	−0.04971 0.1064	−0.03442 0.09976
	*p*-value	0.5613	0.5974	0.2811	0.6519	0.3701	0.5604	0.8505	0.9427	0.5883	0.3734	0.1725	0.9239	0.0235	0.0104	0.3708	0.6804	0.5950	0.7682	0.4844	0.4648	0.9099	0.4789	0.6405	0.7302
Linear	β SE	−0.02710 0.2294	0.3636 0.3003	−0.4014 0.3386	0.1637 0.2122	0.4279 0.3244	−0.1571 0.2728	0.01930 0.1556	0.1336 0.2373	−0.1228 0.2030	0.003109 0.002951	0.006803 0.004292	−0.00061 0.003976	0.02965 0.01220	0.04082 0.01655	0.02089 0.01724	0.03564 0.2862	0.09435 0.4231	0.06718 0.3815	−0.00938 0.01870	−0.00948 0.02598	−0.00578 0.02622	−0.00984 0.02027	−0.00293 0.02794	−0.01262 0.02865
	*p*-value	0.9060	0.2266	0.2363	0.4406	0.1876	0.5650	0.9013	0.5736	0.5452	0.2923	0.1137	0.8789	0.0152	0.0141	0.2263	0.9009	0.8236	0.8603	0.6162	0.7154	0.8258	0.6273	0.9166	0.6598
		**Adjusted model ^3,4,5^**																							
Quartile 1 Median = −1.809		**(Ref)**	**(Ref)**	**(Ref)**	**(Ref)**	**(Ref)**	**(Ref)**	**(Ref)**	**(Ref)**	**(Ref)**	**(Ref)**	**(Ref)**	**(Ref)**	**(Ref)**	**(Ref)**	**(Ref)**	**(Ref)**	**(Ref)**	**(Ref)**	**(Ref)**	**(Ref)**	**(Ref)**	**(Ref)**	**(Ref)**	**(Ref)**
Quartile 2 Median = −0.630	β SE	−0.1622 0.2481	−0.4641 0.3073	0.1158 0.3783	−0.9515 0.7032	−0.9685 1.0724	−0.9390 0.9205	−0.8455 0.5245	−1.2116 0.7953	−0.4926 0.6885	0.01326 0.01025	0.02101 0.01484	0.01012 0.01392	0.02897 0.04094	0.06968 0.05402	0.003959 0.05863	0.7208 0.7511	0.5212 1.0902	0.8175 1.0117	−0.00072 0.05866	0.04457 0.08694	−0.02456 0.07993	−0.00165 0.06456	0.08241 0.09388	−0.04292 0.08926
	*p*-value	0.5133	0.1317	0.7597	0.1763	0.3669	0.3081	0.1072	0.1282	0.4746	0.1958	0.1575	0.4678	0.4794	0.1978	0.9462	0.3376	0.6329	0.4196	0.9902	0.6086	0.7588	0.9797	0.3807	0.6309
Quartile 3 Median = 0.367	β SE	−0.05138 0.2572	0.1094 0.3210	−0.2653 0.3921	−0.4100 0.7205	0.3755 1.0970	−1.2512 0.9455	−0.5400 0.5348	−0.2713 0.8052	−0.8539 0.7058	0.01760 0.01033	0.03426 0.01475	0.003487 0.01428	0.07035 0.04149	0.05348 0.05512	0.07973 0.05913	−0.2508 0.7793	−0.2023 1.1102	−0.02910 1.0755	0.03325 0.05918	0.1630 0.08286	−0.07402 0.08592	0.03778 0.06475	0.2121 0.08928	−0.09819 0.09589
	*p*-value	0.8417	0.7333	0.4990	0.5694	0.7322	0.1862	0.3128	0.7363	0.2268	0.0886	0.0206	0.8072	0.0903	0.3325	0.1781	0.7477	0.8555	0.9784	0.5744	0.0499	0.3895	0.5598	0.0180	0.3065
Quartile 4 Median = 1.627	β SE	−0.06379 0.2710	0.2387 0.3430	−0.3789 0.4077	−0.1990 0.7460	0.4404 1.1387	−0.9106 0.9770	−0.4396 0.5500	−0.3937 0.8245	−0.6105 0.7282	0.007480 0.01061	0.02143 0.01547	−0.00365 0.01452	0.09054 0.04314	0.1463 0.05779	0.03924 0.06192	0.5218 0.8310	0.6096 1.2068	0.7541 1.1187	−0.04283 0.06388	−0.03861 0.09624	−0.04039 0.08593	−0.05409 0.06986	−0.00900 0.1034	−0.06534 0.09521
	*p*-value	0.8139	0.4868	0.3531	0.7897	0.6990	0.3517	0.4243	0.6332	0.4021	0.4811	0.1665	0.8015	0.0361	0.0117	0.5265	0.5302	0.6137	0.5006	0.5028	0.6885	0.6386	0.4389	0.9307	0.4929
Linear	β SE	−0.00700 0.07627	0.1167 0.09747	−0.1327 0.1143	−0.00199 0.2097	0.2405 0.3203	−0.2696 0.2738	−0.08756 0.1545	−0.01362 0.2313	−0.1931 0.2041	0.002315 0.002950	0.006580 0.004281	−0.00142 0.004051	0.02739 0.01206	0.03837 0.01626	0.01612 0.01723	0.05919 0.2340	0.1014 0.3391	0.1344 0.3152	−0.00876 0.01766	−0.00146 0.02556	−0.01473 0.02453	−0.01133 0.01925	0.005503 0.02711	−0.02213 0.02726
	*p*-value	0.9269	0.2319	0.2461	0.9924	0.4531	0.3253	0.5711	0.9531	0.3444	0.4327	0.1250	0.7259	0.0233	0.0187	0.3498	0.8003	0.7651	0.6700	0.6199	0.9546	0.5483	0.5563	0.8393	0.4174

^1^ Median values of C-DII score at each quartile. ^2^ Model includes C-DII score quartiles as fixed effects and compound symmetry matrix structure to model the covariance structure of the repeated measurements for each outcome. ^3^ Models additionally adjusted for the following fixed effects mother’s enrollment in the calcium intervention study, mother years of education at childbirth, child age, pubertal onset, metabolic equivalents, and calories. ^4^ Sex is an additional fixed effect in the adjusted models for the overall sample. ^5^ BMI is an additional fixed effect in the waist circumference models.* *p* < 0.00625.

## Data Availability

Not applicable.

## Supplementary Materials

The following supporting information can be downloaded at: https://www.mdpi.com/article/10.3390/nu14040896/s1, Table S1. Scoring Criteria for Dietary Approach to Stop Hypertension (DASH) Score; Table S2. Scoring Criteria for Alternate Mediterranean Diet (aMedDiet) Score; Table S3. Availability of Children’s Dietary Inflammatory Index (C-DII) Sub-components Used in the Current Study.
