# Supplementary material for: Diet Quality Scores and Cardiometabolic Risk Factors in Mexican Children and Adolescents: A Longitudinal Analysis
Source: Nutrients. 2022 Feb 20;14(4):896. doi: 10.3390/nu14040896 (PMC8878155; doi:10.3390/nu14040896)
Supplement: Supplementary file 1 [file nutrients-14-00896-s001.zip › nutrients-1566050-supplementary.pdf]

Supplemental Table S1. Scoring Criteria for Dietary Approaches to Stop Hypertension (DASH) Score [1].

No	Food groups (gm/day)	Food included	Scoring criteria [1]
**1**	Fruits	All fruits and fruit juices	Quntile 1 = 1 point; Quntile 2 = 2 points; Quntile 3 = 3 points; Quntile 4 = 4 points; Quntile 5 = 1 points
**2**	Vegetables	All vegetables except potatoes and legumes	
**3**	Nuts and legumes	Nuts and peanut butter, dried beans, peas, and tofu	
**4**	Whole grains	Brown rice, whole wheat bread, cooked cereal, whole-grain cereal, other grains, popcorn, wheat germ, and bran	
**5**	Low-fat dairy [2]	Skim milk, yogurt, and cottage cheese	
**6**	Sodium [3]	Sum of sodium content of all foods in FFQ	Quntile 1 = 5 points; Quntile 2 = 4 points; Quntile 3 = 3 points; Quntile 4 = 4 points; Quntile 5 = 1 point
**7**	Red and processed food	Beef, pork, lamb, deli meats, organ meats, hot dogs, and bacon	
**8**	Sweetened beverages	Carbonated and noncarbonated sweetened beverages	

[1] Sex- and age-specific quintiles.

[2] The FFQ did not distinguish between low fat and high fat dairy products, so we included all dairy products in one group

[3] Sodium intake was ranked without age or sex stratification.

Supplemental Table S2. Scoring Criteria for Alternate Mediterranean Diet (aMedDiet) Score [2].

No	Food groups (gm/day)	Food included	Scoring criteria for 1 point
**1**	Fruits	All fruits and juices	Greater than sex- and age-specific median intake
**2**	Vegetables	All vegetables except potatoes	
**3**	Legumes	Tofu, string beans, peas, beans	
**4**	Nuts	Nuts, peanut butter	
**5**	Whole grains	Whole-grain ready-to-eat cereals, cooked cereals, crackers, dark breads, brown rice, other grains, wheat germ, bran, popcorn	
**6**	Fish	Fish and shrimp, breaded fish	
**7**	Ratio of monounsaturated to saturated fatty acids	Sum of monounsaturated fatty acids content of all foods in FFQ/ sum of saturated fatty acids content of all foods in FFQ	
**8**	Red and processed food	Hot dogs, deli meat, bacon, hamburger, beef	Less than or equal to sex- and age-specific median intake

Supplemental Table S3. Availability of Children's Dietary Inflammatory Index (C-DII) Sub-components Used in the Current Study [3].

No	C-DII Sub-components	Availability
**1**	Alcohol (g)	✓
**2**	Vitamin B12 (mcg)	✓
**3**	Vitamin B6 (mg)	✓
**4**	Beta Carotene (mcg)	✓
**5**	Carbohydrate (g)	✓
**6**	Cholesterol (mg)	✓
**7**	Energy (kcal)	✓
**8**	Fat (g)	✓
**9**	Fiber (g)	✓
**10**	Folic Acid (mcg)	✓
**11**	Iron (mg)	✓
**12**	Magnesium (mg)	✓
**13**	Monounsaturated fatty acids (g)	✓
**14**	Niacin (mg)	✓
**15**	Protein (g)	✓
**16**	Polyunsaturated fatty acids (g)	✓
**17**	Riboflavin (mg)	✓
**18**	Saturated Fat (g)	✓
**19**	Selenium (mcg)	✓
**20**	Thiamin (mg)	✓
**21**	Vitamin A (RE)	✓
**22**	Vitamin C (mg)	✓
**23**	Vitamin D (mcg)	X
**24**	Vitamin E (mg)	✓
**25**	Zinc (mg)	✓

References

1. Fung, T.T.; Chiuve, S.E.; McCullough, M.L.; Rexrode, K.M.; Logroscino, G.; Hu, F.B. Adherence to a DASH-style diet and risk of coronary heart disease and stroke in women. *Arch Intern Med* **2008**, *168*, 713-720, doi:10.1001/archinte.168.7.713.
2. Fung, T.T.; McCullough, M.L.; Newby, P.K.; Manson, J.E.; Meigs, J.B.; Rifai, N.; Willett, W.C.; Hu, F.B. Diet-quality scores and plasma concentrations of markers of inflammation and endothelial dysfunction. *Am J Clin Nutr* **2005**, *82*, 163-173, doi:10.1093/ajcn.82.1.163.
3. Khan, S.; Wirth, M.D.; Ortaglia, A.; Alvarado, C.R.; Shivappa, N.; Hurley, T.G.; Hebert, J.R. Design, Development and Construct Validation of the Children's Dietary Inflammatory Index. *Nutrients* **2018**, *10*, 993, doi:10.3390/nu10080993.
